# The Hidden Hazards of Vaping

**DOI:** 10.1002/lary.70595

**Published:** 2026-04-28

**Authors:** Ayaantuu F. Usman, Sobia F. Khaja

**Affiliations:** ^1^ Department of Otolaryngology University of Minnesota Minneapolis Minnesota USA

**Keywords:** motor vehicle collision, penetrating oropharyngeal trauma, vape pen injury

## Abstract

A 31 year old male presented with a penetrating oropharyngeal injury following a motor vehicle collision and was found to have a retained metallic foreign body suspected to be part of a vape pen. The foreign body was abutting the carotid artery and was removed via a combined transoral and transcervical approach. This case highlights a novel mechanism of head and neck trauma and emphasizes the importance of understanding a wide spectrum of unique injuries.

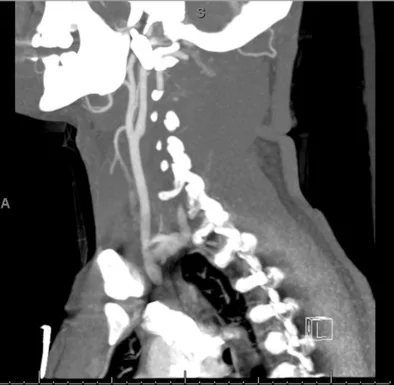

## 1 Introduction

E‐cigarette usage is steadily increasing worldwide, with an estimated 82 million vapers [1]. Numerous studies investigate the harms of e‐cigarettes and motor vehicles from a lens of distraction and possible impairment while driving [2]. While many of the worries deal with lung injuries, cancer risks and public safety concerns, there were no identified reports that investigated trauma caused by these devices during motor vehicle collisions (MVC) and their potential to cause penetrating injuries is overlooked. Here we present a case of a motor vehicle crash‐related penetrating injury caused by a vape pen to highlight the hazards of these devices.

## 2 Case Report

**FIGURE 1:**
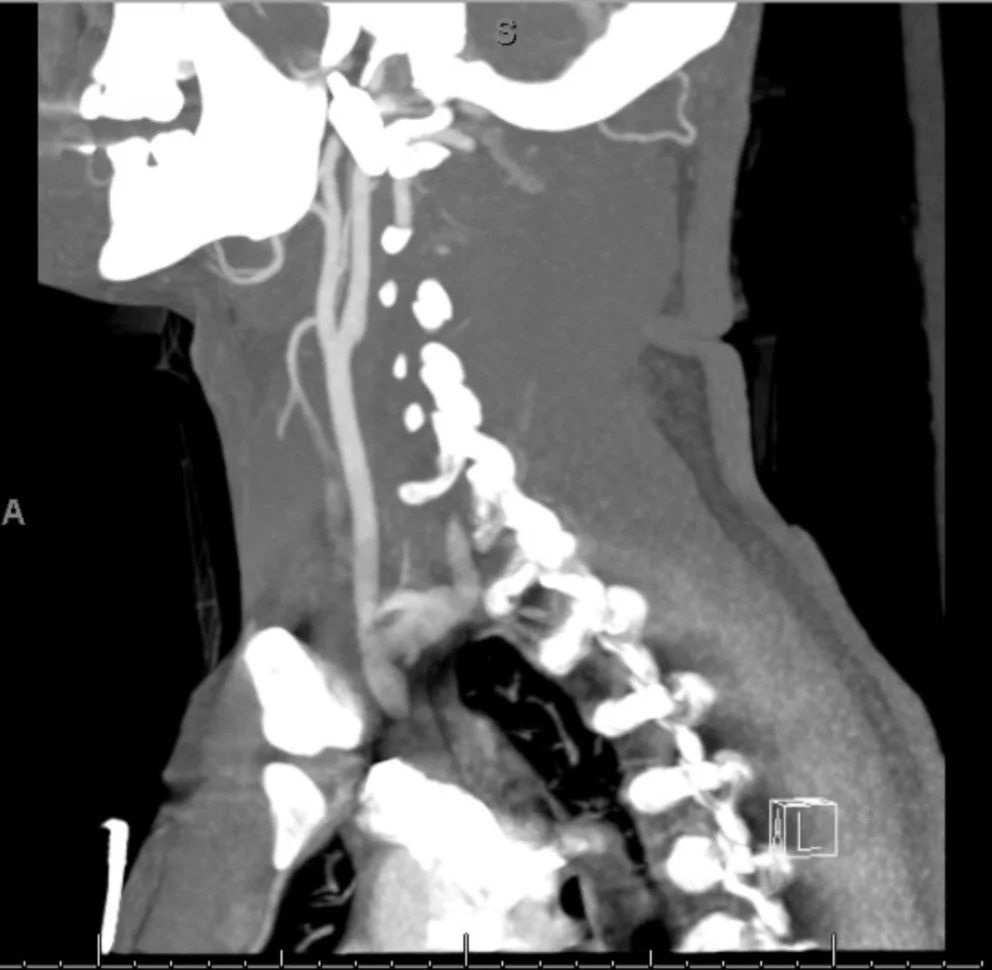
Sagittal CTA neck image showing foreign body in right parapharyngeal space.

**FIGURE 2:**
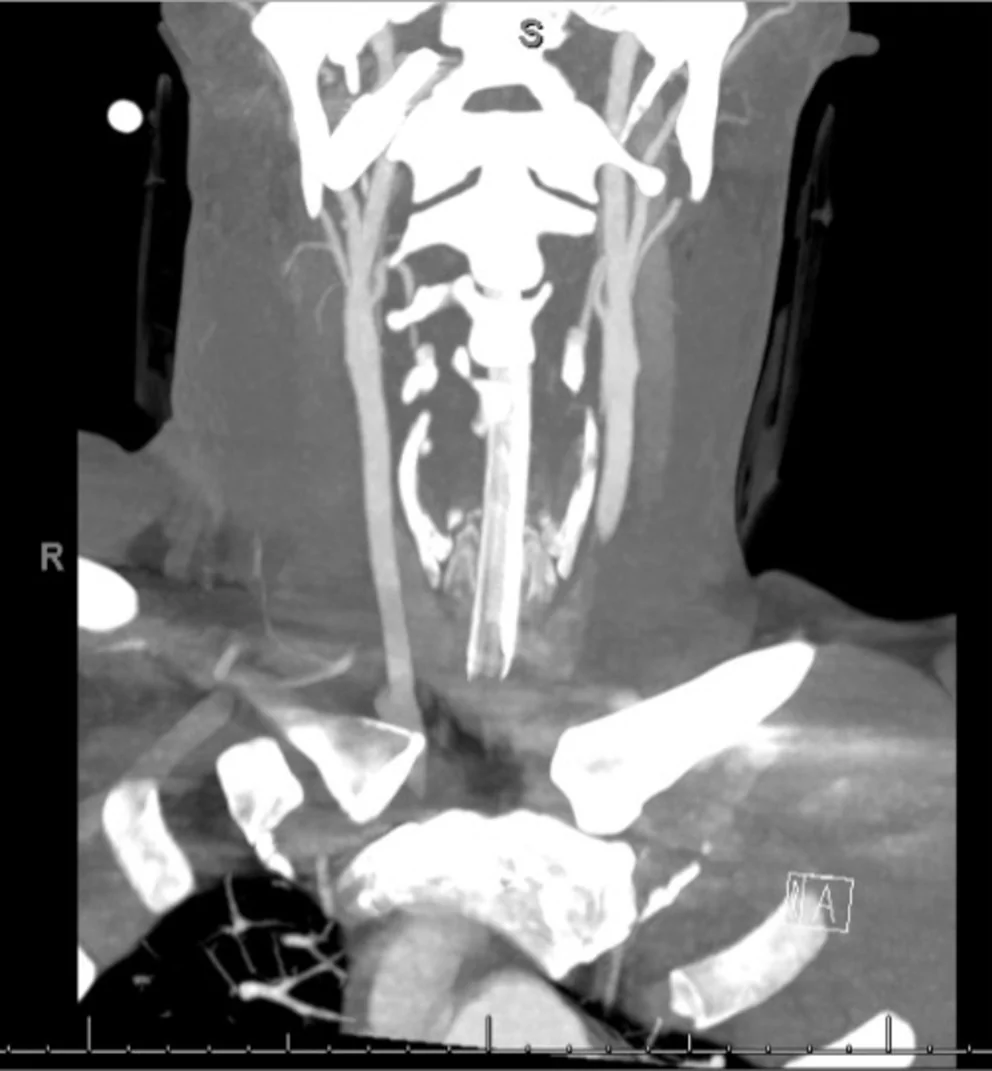
Coronal image of CTA neck showing vape pen touching the carotid artery wall.

**FIGURE 3:**
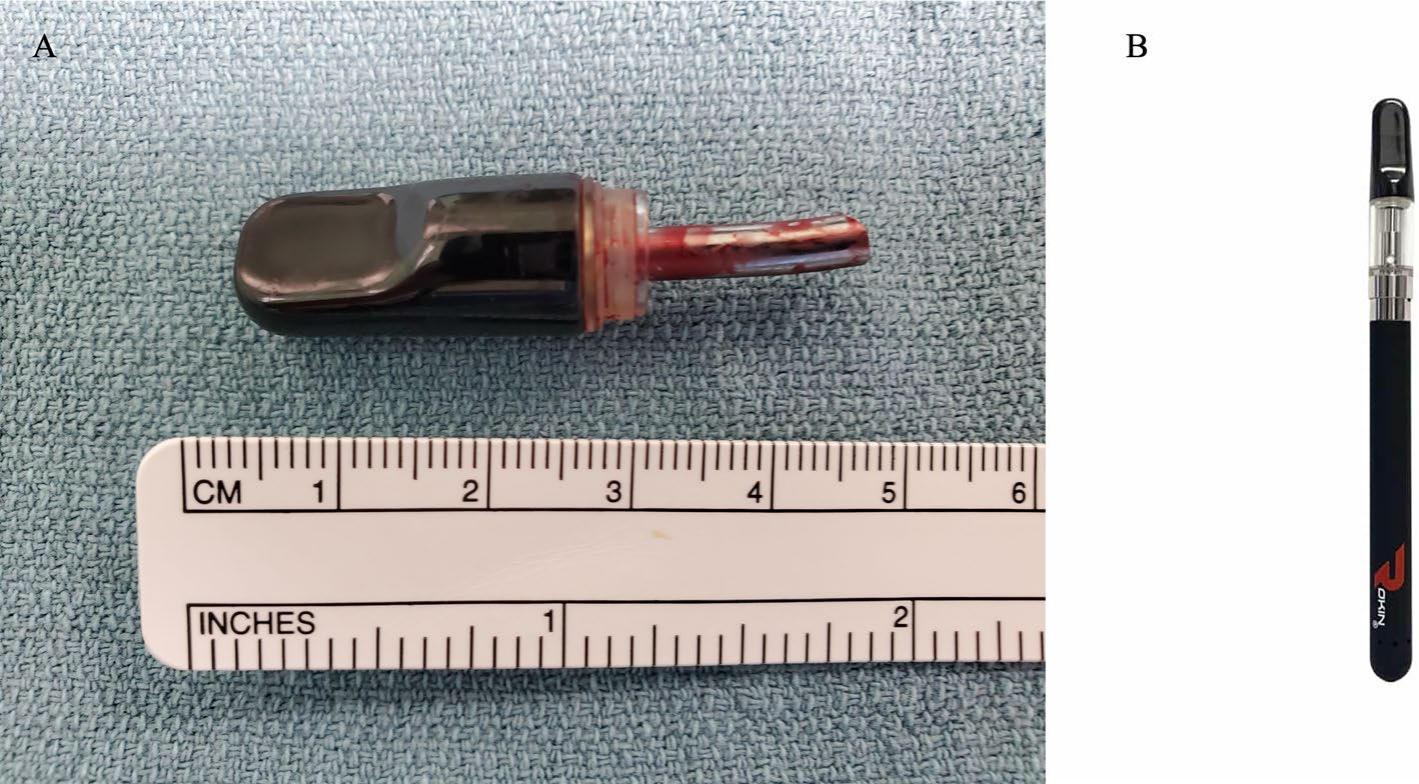
(A) Intraoperative photo of removed foreign body. (B) Representative image of an intact vape pen used for comparison. Image reproduced with copyright permission from Rokin Vapes. [Color figure can be viewed in the online issue, which is available at www.laryngoscope.com]

**FIGURE 4:**
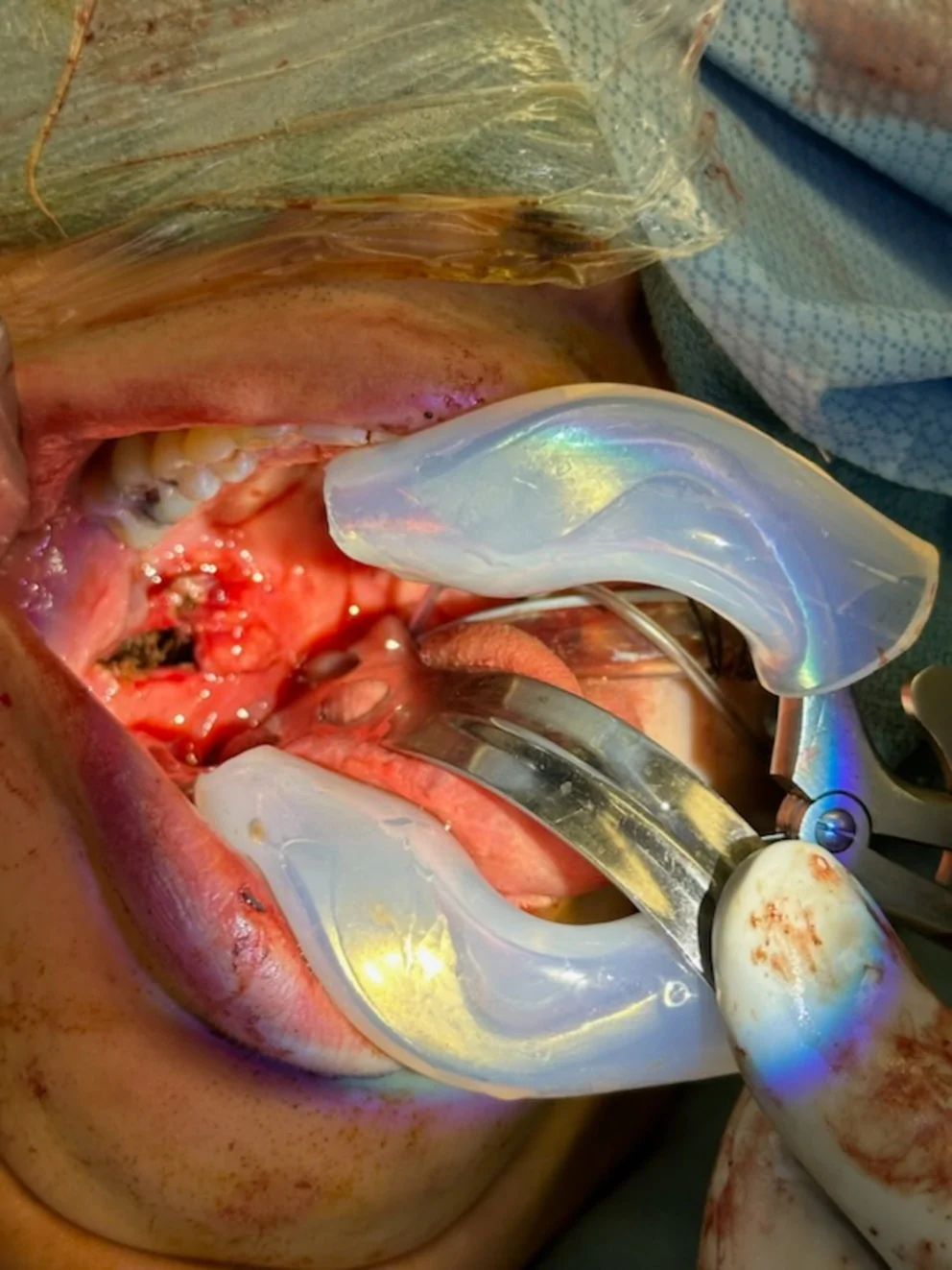
Intraoperative photo of soft tissue injury in oropharynx. [Color figure can be viewed in the online issue, which is available at www.laryngoscope.com]

**FIGURE 5:**
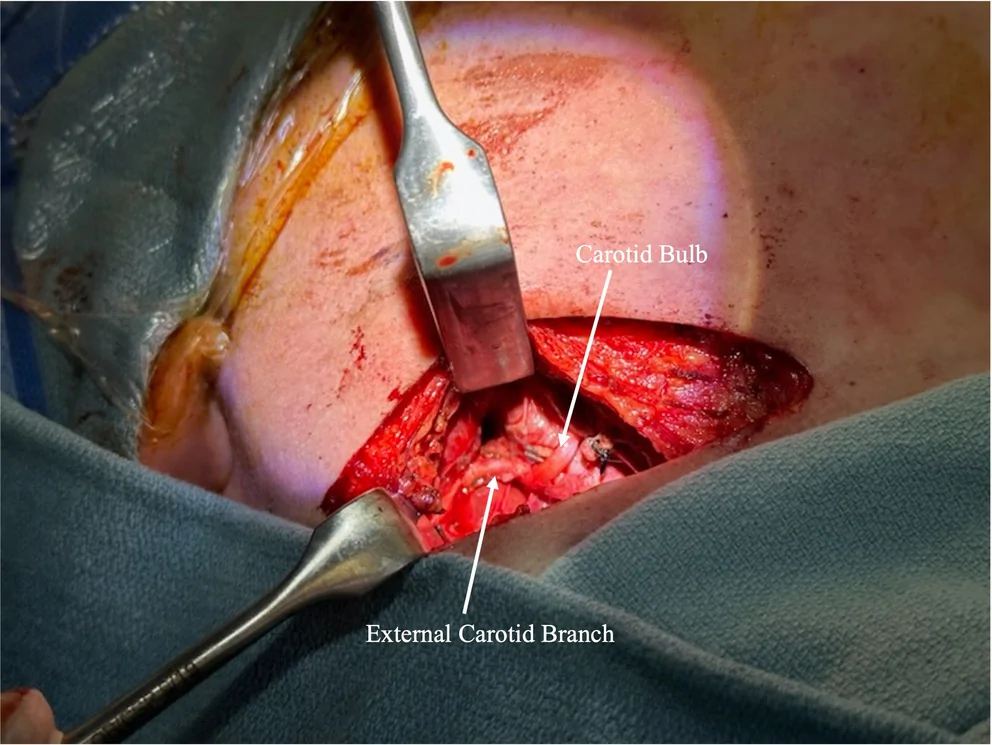
Intraoperative photo of transcervical approach for combined approach removal. [Color figure can be viewed in the online issue, which is available at www.laryngoscope.com]

A 31‐year‐old man presented to the ER following a MVC into a tree at unknown speeds. The patient underwent routine trauma workup upon presentation to the local ER, with his only complaint being throat pain and exam demonstrating a 5 mm soft palate avulsion. On neck CT, he was incidentally noted to have a 4.1 cm long metallic foreign object in the parapharyngeal space. A CTA head/neck demonstrated the metallic object to be abutting the carotid artery, with inability to rule out injury to the vessel wall (Figures 1 and 2). He was transported to the university for multidisciplinary care, where angiography by neuro interventional radiology showed focal compression of the right internal carotid artery (ICA) with partial stenosis, possible dissection without thrombus, and incidental contralateral left carotid‐cavernous fistula. He was taken to the operating room for a combined transoral/transcervical exploration with removal of the foreign body, with control of the vessels and vascular surgery on standby for possible carotid injury management. Intraoperatively, the foreign body was determined to be a broken vape pen (Figure 3) with broken glass present in the oropharynx, with significant soft tissue trauma to the oropharynx and retromolar trigone without carotid injury (Figures 4 and 5). The pharyngeal injury was repaired primarily. The patient presented to the ER 9 days postoperatively following an episode of bleeding after he started vaping and was found to have partial dehiscence of his oropharynx wound, which was attempted to be partially repaired at bedside, with the remainder left for secondary intention. He was NPO with a nasogastric tube in place for approximately 1 month postoperatively while healing.

## 3 Discussion

Penetrating oropharyngeal trauma is a potentially life‐threatening injury with most documented cases dealing with toothbrushes, cylindrical toys and chopsticks [3]. As e‐cigarette use becomes increasingly more common, reports regarding injuries from these devices have not been documented. The existing literature primarily focuses on complications from these devices such as burns, explosions, and inhalation injury [4]. However, trauma related to the physical device during accidents has not been investigated.

The evaluation of penetrating injuries to the head and neck begins with prompt assessment of airway and hemodynamic stability [5]. Patients who are unstable and demonstrate any of the “hard signs”—such as shock, wound bubbling, stridor or hoarseness—should undergo immediate surgical exploration [5]. In contrast, stable patients without hard signs should undergo CT with angiography to evaluate for vascular injury. If the airway is threatened but anatomic structures are preserved, intubation whether standard intubation or fiberoptic intubation versus tracheostomy is recommended [5]. If vascular injury is suspected, vascular surgery consultation is warranted, and if a common or ICA injury is identified during exploration, repair of the artery is prioritized over artery ligation [5].

This case is unique in that it describes an oropharyngeal injury sustained from an e‐cigarette following a MVC. It highlights a mechanical injury where the vape pen likely fractured upon impact and penetrated the oropharynx, resulting in the focal compression of the ICA. This case emphasizes how e‐cigarettes can act as foreign bodies in traumas, specifically in settings such as driving. Additionally, the clinical significance of the location of the penetrating trauma placed the patient at risk for a carotid injury. Due to this presentation, an angiogram was required to assess for such injury prior to urgent removal of the foreign body in the operating room with preparation for possible carotid management.

The importance of proper identification and workup of penetrating trauma in the head and neck is crucial for reducing morbidity and mortality. Careful assessment of the patient's presentation is essential to avoid unnecessary interventions while making sure to promote a timely diagnosis and treatment.

Although this report features a single patient, it stresses the importance for physicians to be aware of novel injury mechanisms that can be caused by e‐cigarettes. Further research on e‐cigarette structure and variations is needed as variation in head and neck anatomy may change injury patterns.

## 4 Conclusion

We present a rare case of a penetrating oropharyngeal injury from a broken vape pen following a MVC resulting in ICA compression and stenosis. This stresses the importance of understanding the wide spectrum of unique injuries that vape pens can cause, which are important to consider in trauma cases and ensuring appropriate workup/management of these injuries.

## Funding

The authors have nothing to report.

## Conflicts of Interest

The authors declare no conflicts of interest.

## Data Availability

Data sharing not applicable to this article as no datasets were generated or analysed during the current study.
